# Senotherapeutics to Counteract Senescent Cells Are Prominent Topics in the Context of Anti-Ageing Strategies

**DOI:** 10.3390/ijms25031792

**Published:** 2024-02-01

**Authors:** Anna Calabrò, Giulia Accardi, Anna Aiello, Calogero Caruso, Damiano Galimberti, Giuseppina Candore

**Affiliations:** 1 Laboratory of Immunopathology and Immunosenescence, Department of Biomedicine, Neurosciences and Advanced Diagnostics, University of Palermo, 90134 Palermo, Italy; anna.calabro@unipa.it (A.C.); giulia.accardi@unipa.it (G.A.); anna.aiello@unipa.it (A.A.); giuseppina.candore@unipa.it (G.C.); 2 Italian Association of Anti-Ageing Physicians, 20133 Milan, Italy; damiano.galimberti@gmail.com

**Keywords:** ageing, immunosenescence, immunotherapy, senescence, senolytics, senomorphics

## Abstract

Cellular senescence is implicated in ageing and associated with a broad spectrum of age-related diseases. Importantly, a cell can initiate the senescence program irrespective of the organism’s age. Various stress signals, including those defined as ageing hallmarks and alterations leading to cancer development, oncogene activation, or loss of cancer-suppressive functions, can trigger cellular senescence. The primary outcome of these alterations is the activation of nuclear factor (NF)-κB, thereby inducing the senescence-associated secretory phenotype (SASP). Proinflammatory cytokines and chemokines, components of this phenotype, contribute to chronic systemic sterile inflammation, commonly referred to as inflamm-ageing. This inflammation is linked to age-related diseases (ARDs), frailty, and increased mortality in older individuals. Additionally, senescent cells (SCs) accumulate in multiple tissues with age and are believed to underlie the organism functional decline, as demonstrated by models. An escalating effort has been dedicated to identify senotherapeutics that selectively target SCs by inducing apoptosis; these drugs are termed senolytics. Concurrently, small molecules that suppress senescent phenotypes without causing cell death are known as senomorphics. Both natural and synthetic senotherapeutics, along with immunotherapies employing immune cell-mediated clearance of SCs, currently represent the most promising strategies to combat ageing and ARDs. Indeed, it is fascinating to observe that information regarding the immune reaction to SCs indicates that regulation by specific lymphocyte subsets, elevated in the oldest centenarians, plays a role in attaining extreme longevity. Regardless, the application of methods already utilized in cancer treatment, such as CAR cells and monoclonal antibodies, broadens the spectrum of potential approaches to be utilized.

## 1. Introduction

Reaching the age of 100 or more is a rare occurrence, but its frequency is gradually increasing in tandem with the rising life expectancy of the world population. Moreover, centenarians serve as an example of healthy ageing, particularly for those dedicated to the study of longevity [1]. Most of these remarkable individuals are classified as “escapers”, demonstrating the ability to evade the onset of pathological phenomena, commonly referred to as ARDs. These diseases include conditions such as Alzheimer’s, cardiovascular issues, diabetes, and osteoarthritis. Meanwhile, the remaining segment of this older population can be categorized as “delayers” or “survivors”, capable of postponing the onset of diseases or overcoming them, respectively, without experiencing severe short- or long-term consequences [2]. Longevity studies aspire to facilitate the process of ageing as successfully as possible, contributing to the extension of healthspan. As emphasized by the United Nations, in tandem with the global increase in population, there is a pressing need to enhance the health, social, and psychological well-being of older individuals. This goal aims not only to prolong life but also to enrich the quality of life by “adding life to years” in addition to “adding years to life” [3]. ARDs arise from a decline in function or a disturbance of homeostatic conditions, inherent to the ageing process. Therefore, ageing is influenced by a complex interplay of conditions, wherein ARDs manifest as outcomes but do not constitute the ageing process itself [4]. At the core of the ageing process, there exist molecular phenomena that govern the emergence of changes characteristic of ARDs. These changes have been succinctly outlined as the “hallmarks of ageing”, a concept introduced by López-Otín in 2013 [5] and subsequently reviewed in 2022 [6]. These hallmarks are categorized into primary, integrative, and compensatory groups, encompassing genomic instability, telomere shortening, epigenetic alterations, mitochondrial dysfunction, loss of proteostasis, deregulation of nutrient sensing, altered intracellular communication, stem cell exhaustion, and cell senescence. To this list, recent additions include microbiota alteration, inflammation, compromised autophagy, splicing dysregulation, and altered mechanical properties [6]. Each of these hallmarks holds the potential as a target for prospective therapies. Notably, cell senescence represents an integrative hallmark since its initiation is contingent on the disruptions determined by the other hallmarks, a topic explored further in the following discussion.

Medications designed to safeguard against ARDs and enhance overall health and longevity are referred to as geroprotector drugs [7]. These drugs target ageing processes, including chronic inflammation, oxidative stress, cellular senescence, and metabolic dysfunction. They aim to promote glucose regulation, enhance autophagy, reduce inflammation, and improve mitochondrial function. While the efficacy and safety of such therapies in humans are still under investigation [8], this review will specifically delve into the role of senotherapeutic agents, which fall under the class of geroprotectors.

Senotherapeutic agents encompass natural compounds, synthetic molecules, or drugs that act on SCs. The class of compounds that act by eliminating SCs by regulating the apoptotic process are specifically defined as senolytics. Senotherapeutic agents induce either the elimination, reduction, or limitation of the secretory capacity of these cells, identified by the term SASPs, defining the class of compounds called senomorphics. SCs tend to accumulate in organs as individuals age, leading to a disruption of their functions and eventual clinical manifestation as diseases. It is noteworthy, however, that SCs play a positive role in countering cancer (considered an ARD), aiding in wound healing irrespective of age, and contributing to embryogenesis [9].

## 2. Cell Senescence

Cell senescence is one of the hallmarks identified by López-Otín and his colleagues in their description of the ageing process [5]. However, it is crucial to recognize that senescence is not solely a consequence of ageing; rather, it is a physiological process that unfolds throughout the entire lifespan.

In 1961, Leonard Hayflick and Paul Moorhead introduced the concept of the limit of cell replication, determining that cells cease to replicate after a certain number of divisions due to the onset of damage [10]. Upon reaching this limit, cells arrest the cell cycle and enter a steady state, becoming resistant to the apoptosis process. This resistance is achieved by upregulating the expression of antiapoptotic genes [11,12], resulting in a state known as cell senescence.

It is essential to distinguish the concept of senescence from that of quiescence and terminal differentiation. Quiescence is a reversible condition, achievable through the administration of mitogens, while terminally differentiated cells signify the cessation of cell function and growth [13]. In the onset of senescence, cell replication halts when irreversible DNA lesions occur, triggering the activation of cell cycle inhibitors (p53/p21, p19, p16INK4-RB) [13]. DNA damage activates the p53-related DNA damage response (DDR) system, involving ataxia telangiectasia mutated (ATM) and ATM Rd3-related [13]. p53 induces cell cycle exit by expressing the cyclin-dependent kinase inhibitor, p21, which inhibits the phosphorylation of *retinoblastoma* (*RB*) gene [13]. Similarly, there is an increased expression of p16, an inhibitor of CDK4/6 [14].

Cell damage, which leads to senescence, can result from various factors, such as epigenetic changes, telomere shortening and associated pathways, alterations in molecular expression, changes in macromolecular structures, and metabolic shifts, as well as modifications in secretory phenotypes [12]. Genomic instability within the senescent process encompasses laminin B degradation and impaired chromatin regulation [14].

The role of telomere shortening in determining cellular senescence is still much debated. Although the process of shortening is generally cited as one of the main causes of senescence, there is also the activation response to DDR at the telomere ends, which leads to the formation of telomere-associated DDR foci or telomere-induced DNA damage foci, which are considered as markers of cellular senescence in cells and tissue cultures [15]. Indeed, it is the formation of the signalling of DDR at telomere loci that may contribute to cell cycle disruption [16]. Ageing mouse models deficient in telomerase, such as G3 terc^−/−^, have shown an accumulation of telomere-associated DNA damage foci with age [17]. This type of mouse model makes it possible to study the effects of telomere shortening on the induction of associated senescence and ARDs, and in this case to evaluate the effect of the contribution of telomere shortening itself or the resulting ARD mechanisms.

Mitochondrial dysfunction is another mechanism inducing cellular senescence, leading to increased production of reactive oxygen species (ROS)/reactive nitrogen species and subsequent damage to the macromolecular structures of cells [11]. Elevated oxidative stress results in damage to proteins and molecular structures. This, coupled with increased expression of mammalian target of rapamycin (mTOR), contributes to the alteration of nutrient sensing. The decline in sirtuins and poly-ADP ribose polymerase further increases SASPs and activates NF-κB [11].

mTOR induction concurrently inhibits the autophagy process, preventing the elimination of macromolecular structures damaged by oxidative stress. Additionally, SC, although nonproliferative, exhibits metabolic activity characterized by a shift towards glycolysis, reduced fatty acid oxidation, and increased ROS production [18]. The secretory activity of these cells results in SASPs, hindered by elevated oxidative stress and DNA damage linked to NF-κB activation [11].

Notably, NF-κB1, particularly, plays a central role in this process and may contribute to cellular senescence induction [19,20]. Cyclin-dependent kinases (CDKs) are implicated in SASP production and the spread of cell senescence. CDK6 activity is linked to the transcription of NF-κB and STAT3, key mediators involved in the expression of proinflammatory proteins constituting SASPs. CDK6 induces the expression of p16 and recruits p65, a subunit of NF-κB, activating it [21]. Therefore, the production of SASPs is intricately connected to induced cell damage, leading to cell senescence through NF-κB and the modulation of CDKs and cell cycle-regulating proteins in a dense communication network.

The release of SASPs plays a paracrine role, inducing senescence in neighbouring cells and contributing to tissue dysfunction and the onset of ARDs. Indeed, most SCs are characterized by a proinflammatory, profibrotic, and proapoptotic phenotype, and their persistence in the environment leads to the widespread propagation of senescence [22]. A smaller subset of SCs exhibits less profibrotic activity, promoting tissue regeneration through the secretion of growth factors such as platelet derived growth factor and vascular endothelial growth factor [22].

Furthermore, the inability of stem cells to replace SCs, due to stem cell exhaustion, diminishes the potential for restoring the functions of damaged tissues. This, coupled with the dysfunction of the immune system in eliminating SCs and reinstating the balance between proliferative and SCs, is influenced by immunosenescence [23,24].

### 2.1. SCs as Positive Effectors

In discussing the activity of SCs, it is crucial to underscore that their primary role is not inherently negative in a physiological context and during early stages of life. SCs release growth and proapoptotic factors in response to transient cellular damage, exerting a positive influence on nearby cells. This is linked to the clearance of new SCs, promoting immune cell recruitment [25]. The chemotactic activity is part of the immunomodulatory properties of SASPs. Notably, SASP components like IL-8, tumour necrosis factor (TNF)-α, and interleukin (IL)-1 facilitate the recruitment of macrophages, monocytes, and dendritic cells [25].

Similarly, SASPs and SCs can act to inhibit the immune response, creating a form of immune evasion through the upregulation of human leukocyte antigen (HLA)-E, which inhibits CD8+ T cells and natural killer (NK) cells [24,25]. Additionally, the involvement of the immune system, particularly the induction of senescence in immune cells, seems to play a causative role in organismal ageing. To define the contribution of immune ageing to overall organism ageing, the *Ercc1* gene, which encodes a crucial DNA repair protein, was selectively deleted in hematopoietic cells of mice. This increased DNA damage, due to impaired repair, leads to senescence specifically in the immune system. The transgenic mice remained healthy until early adulthood, displaying a premature onset of immunosenescence similar to changes observed during ageing in wild-type mice. Notably, nonlymphoid organs also exhibited an increase in SCs and damage, suggesting that immune ageing “promotes” systemic ageing. Transplanting young immune cells attenuated this process of systemic ageing. This study, utilizing a transgenic murine model where the mouse immune system is impaired, demonstrates on one hand that the immune system is responsible for eliminating SCs. On the other hand, it indicates that SCs are responsible, or at least contribute significantly, to organismal ageing [26,27].

In a physiological environment and during young ages, SCs play a crucial role in embryogenesis and wound repair [28,29,30]. In both these contexts, the presence of SCs in the tissue microenvironment is transient, presumably to prevent the detrimental effects associated with persistent SCs [31]. Conversely, in the context of tissue ageing, the effects of SASPs may be decidedly unfavourable. SCs accumulate in ageing tissues, and their removal appears to reduce tissue dysfunction, potentially contributing to ARDs such as arthritis, atherosclerosis, and neurodegenerative diseases [32,33,34,35,36,37]. Understanding the context dictated by cellular senescence is crucial, given the role of SCs in tumorigenesis.

### 2.2. Role in Tumorigenesis

In the 2000s, van Deursen [38] embarked on a venture with the objective of engineering mice to investigate cancer development and progression. This led to the creation of an animal model of senescence by knockdown of BubR1, a protein crucial for ensuring the proper segregation of chromosomes during cell division. Over a few months, these mice exhibited traits typical of aged mice, including cataracts and organ failure.

In response to replicative stress, cells were hypothesized to undergo senescence as a protective mechanism against malignant transformation, earning them the name “zombie cells”. To elucidate this phenomenon, it is essential to highlight the shared characteristics between SCs and tumour cells. Notably, SCs exhibit molecular features that overlap with those found in cancer cells. For instance, the epigenetic modifications observed during senescence bear a resemblance to those encountered in the context of cancer development [39]. This implies that senescence, typically regarded as a preventive mechanism against cancer, might paradoxically play a role in initiating cancer. The escalation of cellular senescence with age correlates with a simultaneous rise in cancer incidence. From this standpoint, it appears that SCs re-entering the cell cycle, spurred by appropriate stimuli, may face an elevated risk of transforming into cancerous cells [39].

Another contributing factor could be the induction, facilitated by a paracrine mechanism, of a carcinogenic phenotype in neighbouring cells. Regarding this theory, there is a consensus among many researchers that SCs themselves may not directly transform into cancer cells. Instead, they may play a role in promoting cancer formation in contexts driven by chronic inflammation accompanying the ageing process, defined as inflamm-ageing [38]. This inflammation, which is promoted and continued by a multiplicity of exogenous and endogenous stressors in older individuals, is favoured by the accumulation of SCs during ageing and is perpetuated by both the systemic spread of SASPs and the associated SASP-related effects [27,40].

On the contrary, the induction of cellular senescence in cells experiencing irreversible DNA damage or damage to oncogenic genes, such as K-Ras, has been demonstrated to halt cancer transformation [39]. Adding to these theories, the overexpression of oncogenic signals is a common feature between SCs and cancer cells [39]. This results in the upregulation of genes like *p16* and *p53*, subsequently triggering the induction of a senescent phenotype. Thus, the cell cycle arrest mechanism involving the upregulation of p53/p21 plays a role in inhibiting neoplastic promotion. Indeed, inducing p53 expression in p53-null cells has been shown to promote senescence [39].

However, it is crucial to note that the production of SASPs can stimulate the proliferation of neighbouring cells, thereby increasing the likelihood of DNA mutations. These mutations, in turn, contribute to tumorigenesis. The removal of p16-positive cells from ageing mouse models has been observed to increase cancer-free survival [39]. In conclusion, the question regarding the role of SCs in tumorigenesis remains unresolved.

### 2.3. SCs Molecular Biomarkers

For many years, the physiological relevance of senescence was ignored, and cellular senescence was considered an artifact associated with tissue culture. The contrast with apoptosis (a parallel cellular fate that plays important roles in health and disease) was significant. The pathophysiological implications of apoptosis were evident from the 1990s and 2000s, thanks in part to the clarity provided by markers such as the induction of caspase activity. During that time, the physiological relevance of senescence was still the subject of heated debates, due to the difficulties in identifying SCs in vivo. In recent years, especially thanks to the murine model studied by Yousefzadeh et al. [26] understanding of the role of cellular senescence in organismal ageing has begun to emerge [41]. The emerging and increasingly role of cellular senescence in age-related pathological and clinical manifestations necessitates the identification of specific markers enabling the identification of SCs in vivo. The methods available for detecting and confirming senescence-associated biomarkers span from traditional imaging techniques, such as in situ hybridization, immunohistochemistry, and molecular analysis, to in vivo modelling [41,42]. Mouse models serve as valuable tools for understanding the role of cell cycle checkpoints in the induction of senescence and as potential biomarkers. The role of p16 in the induction of SCs and senescence-associated tumorigenesis remains somewhat unclear, but it can be considered a biomarker of senescence and ageing. Its expression increases in response to cell damage, targeting the cyclin-dependent kinase CDK4/6 and simultaneously inhibiting the phosphorylation of RB, thereby impeding the progression of the cell cycle from G1 to S phase. Furthermore, p16 expression is notably elevated in the context of senescence associated with ageing, contrasting with its absence in young cells. As a general biomarker of ageing, p16 expression appears to rise in response to ageing-inducing stimuli like cigarette smoking, sedentariness, and drug treatments, as observed in vitro, where both mRNA and protein levels of p16 were predominantly associated with T cells isolated from peripheral blood samples. This underscores that p16 levels are higher in older subjects compared to younger ones [14,43]. Despite these characteristics, the expression of p16INK4 is not specific to SCs and is not uniformly expressed in all SCs [42]. Consequently, p16 may be considered a marker of senescence when upregulated alongside an increased presence of senescence-associated β-galactosidase [44,45]. In vitro, SCs are, indeed, characterized by high levels of lysosomal β-galactosidase (β–Gal) activity, known as Senescence Associated–β–Gal (SA–β–Gal). Damaged or diseased tissues generally contain SA–β–Gal-positive cells, while normal and healthy tissues are negative for this marker [36,44,45,46,47,48]. Alterations in mitochondria and lysosomes can also be considered as markers of senescence to be taken into account. SCs are characterized by an increase in the number of mitochondria and lysosomes, which, however, present functional alterations. The former are in fact involved in the increased production of ROS, in alterations in the molecules involved in energy metabolism and in the electron transport chain (ATP synthesis), which in turn are linked to the autophagy and proteostasis alteration mechanisms typical of cellular senescence [42]. Inhibition of the electron transport chain is associated with an imbalanced distribution of energy molecules (AMP, ADP, and ATP) and the coenzymes involved in the process (NAD^+^/NADH), impacting poly-ADP ribose polymerase and sirtuins—mechanisms that influence NF-κB activation. Mitochondrial clearance has been observed to have an impact on SCs and the production of SASP. On the other hand, lysosomes play a role in eliminating damaged cellular components, and an increased number of lysosomes is associated with the accumulation of damaged organelles during senescence induction [42]. SA–β–Gal is not the sole marker linked to lysosomal disruption; the accumulation of lipofuscin in lysosomes, which correlates with the stimulation of the antiapoptotic factor B-cell lymphoma (Bcl)-2, can also be defined as a marker of senescence [42].

Other markers of senescence need consideration. For instance, the role of p53 in cellular senescence is pivotal. Studies involving human fibroblasts and mammary cells with downregulated p53 expression reveal a concurrent upregulation of p16, promoting the induction of senescence. However, it remains uncertain whether this alone is adequate to induce a senescent phenotype [39]. On the other hand, p19 seems to function as a regulator of cellular senescence [39]. Ablation of p19 in BurbR1H/H mouse models results in the deterioration of conditions related to cellular senescence, while the elimination of p21 is associated with a deceleration of the senescence process and a reduction in tissue dysfunctions [39]. The role of p21 is also connected to another protein involved in the regulation of cell cycle arrest and, consequently, the initiation of senescence, RB. The heightened presence of CDK inhibitors such as p21 and p16 results in the phosphorylation of RB. This phosphorylation prevents RB from inhibiting E2F activation, thus promoting cell cycle progression and ultimately leading to the onset of cellular senescence [42]. The regulation of the expression of these biomarkers depends on post-transcriptional changes induced by microRNA and long noncoding RNA [42].

At the molecular level, the expression of SASPs is also influenced by DNA damage, chromatin changes, and the senescence-associated hallmarks mentioned earlier. In this context, alterations in heterochromatin foci are considered characteristic of SCs and should be considered. One of these changes involves LINE-1 retrotransposons that activate the type I interferon (IFN) response, contributing to the definition of SASPs. This activation, coupled with the downregulation of lamin B1, correlates with the determination of the epigenetic profile of SCs through cGAS-STING and IFN activity [42].

### 2.4. Senescence Cell Antiapoptotic Pathways (SCAPs)

SCs exhibit resistance to apoptotic stimuli, which typically arise at the onset of irreversible damage to the cell. This resistance is attributed to the overexpression of antiapoptosis-related markers, including Bcl-2 family proteins, p53, serpin, heat shock proteins, Akt, metabolic pathways, and tyrosine kinases [22]. Targeting specific senescence markers such as SCAPs, increased lysosomal activity, and elevated production of SA–β–Gal could potentially facilitate the interruption of senescence processes.

Several mouse models have been developed to explore the modulation of cellular senescence and its impact on the positive mechanisms exerted by SCs. One notable model is the INK–ATTAC, which incorporates a suicide gene into the promoter region of the *p16INK4* gene, upregulated in SCs. However, in certain SCs, the network of SCAPs is highly complex, making it challenging to achieve the desired senolytic effect by targeting a single pathway. Additionally, the expression of SASPs and SCAPs varies among different SCs. Therefore, a senolytics that effectively targets one type of SC may not have the same impact on another, as discussed in the following sections.

### 2.5. SASPs

The induction of cell senescence is driven by a collection of cellular damages associated with the hallmarks of ageing. Senescence, in turn, is an integrative hallmark that encompasses and arises from various factors. DNA damage, the initiation of double-strand breaks, mitochondrial dysfunctions, and loss of proteostasis collectively contribute to the initiation of the production of SASPs [11]. The SASP defines the specific characteristics of a cell undergoing senescence, with certain mediators prevailing over others and contributing to the delineation of a distinct senescent phenotype. This prevalence is contingent upon the stimulus and, consequently, the specific context [22]. This diversity is also evident at the transcriptomic level. The expression of the majority of SASPs is regulated by transcription factors such as p53, NF-κB, Janus kinase (JAK)-STAT, and GATA4 [24]. For instance, the transient expression of SASPs, associated with NF-κB transcription, induces keratinocyte regeneration in vivo, elevating the population of stem-related cells. However, chronic stimulation leads to the impairment of this regenerative process [24]. Thus, the study of the molecular aspect of SCs can open to discovering other senescent-associated markers [24]. Certain SASPs encompass proinflammatory factors (such as cytokines, and chemokines), growth factors, metalloproteases, extracellular vesicles, and proapoptotic and profibrotic factors, albeit in varying proportions among SCs [11,22]. Notably, IL-6 and the C-X-C motif chemokine ligand 8 (CXCL-8) are commonly identified as SASP biomarkers in most, though not all, SCs. SASP likely contributes to the ability of SCs to influence the differentiation of neighbouring cells, playing another crucial role when these transient SCs need to be cleared through immune-mediated mechanisms [28,49,50,51,52,53]. 

In this context, a crucial question arises concerning the characteristics of secondary SCs induced by the paracrine effects exerted by SASPs. Are these secondary SCs dynamically, molecularly, and phenotypically identical to the primary SCs? The phenotype and secretory patterns of SCs are dynamically mutable, especially when exposed to other stimuli, such as damage-associated molecular patterns and pathogen-associated molecular patterns. SCs exhibit a form of plasticity, allowing them to respond to various stimuli after the initiation of the senescent phenotype [23]. For instance, stimuli like lipopolysaccharide (LPS) or spike protein 1 (S1) of SARS-CoV-2 could contribute to elevated SASP levels in older mice. This may lead to the exacerbation of inflammatory conditions, ultimately resulting in cytokine storms and multiorgan failure [54]. Camell et al. presented these findings based on human senescent endothelial cells and an aged mice model [54]. SCs treated with LPS and the S1 antigen demonstrated an increase in cytokines and inflammation mediators. The secretome of these cells led to an enhanced expression of virus entry proteins and a reduction in antiviral effectors. In a parallel observation, aged mice treated with a virus similar to SARS-CoV-2 exhibited an elevation in the senescence rate and the release of SASP. The administration of senotherapeutic agents resulted in a reduction in these effects, highlighting the pivotal role of senescence and its mediators in infections in advanced age [54].

Moreover, SASP makes potent paracrine and autocrine activities to SCs, exhibiting both positive and deleterious effects contingent upon the cellular context [23]. It becomes, at least partially, responsible for reshaping the microenvironment of ageing tissues, thereby contributing to structural and functional degradation and inflamm-ageing [11,55,56]. In the context of the remodelling of the extracellular environment, specific components of SASPs contribute to alterations in the extracellular matrix microenvironment (ECM), subsequently causing dysfunctions typical of SCs. Examples of SASPs carrying out this function include the upregulation of certain metalloproteases, which then initiate matrix degradation, or the modified expression of ECM components like collagen, fibronectin, or proteoglycans [57]. Inflamm-ageing assumes a central and contextual role in the ageing process, playing a role in ARDs such as osteoarthritis, osteoporosis, fibrotic diseases, cardiovascular disturbances, and diabetes. In these conditions, the accumulation of SCs and their secretome exacerbates the existing conditions. As ageing progresses, inflamm-ageing, coupled with immunosenescence, leads to the establishment of an immunosuppressive environment, hindering the inflammatory status in older individuals. SASP, in particular, seems to play a predominant role in exacerbating chronic inflammation in older people, inflamm-ageing, contributing to exacerbating the effects of cellular senescence on the onset of ARDs. SASPs also play a role in recruiting immune cells, including T cells, NK cells, macrophages, and neutrophils, for the transient elimination of damaged cells [40]. However, as ageing advances, the heightened presence of SCs and SASPs leads to the recruitment of cells that lose their functions due to immunosenescence, resulting in impaired phenotypes and reduced numbers [11]. Additionally, SASPs disrupt intercellular communication and impede tissue regeneration after damage. They play a pivotal role in the initiation of fibrotic diseases such as liver fibrosis and idiopathic pulmonary fibrosis. In conditions like osteoarthritis, osteoporosis, and sarcopenia, treatment with senolytic agents also impacts SASP levels. The use of senolytics in diseases such as diabetes and cardiovascular pathologies has been shown to improve health conditions [11].

## 3. Senotherapeutic Drugs

Among the most studied anti-ageing strategies, there are senotherapeutic drugs, for which clinical trials have already been organized.

Senotherapeutic drugs may be compounds, drugs that act to increase the healthspan of older people by acting on the molecular and clinical manifestation of ageing determined by senescence [58]. The use of senotherapeutic agents has been developing in the last years thanks to their action in the efficiency of transplantation in animal models and humans and the reduction in mortality in aged mice models infected with SARS-CoV-2 [54]. The studies about senotherapeutic agents are incomplete, and there is an absence of standardized guidelines related to the use of different compounds in the treatment and the limitation of cell senescence. 

Senotherapeutic drugs are categorized into two distinct classes: senolytics, which selectively target and eliminate SCs; and senomorphics, which regulate the functions and morphological characteristics of SCs to resemble those of youthful cells or hinder the transition of young cells into SCs within tissues. In addition, immune system mediators responsible for clearing SCs are also discussed (Table 1) [59].

### 3.1. Senolytics

Senolytics are a class of drugs that selectively target and eliminate SCs by exploiting their unique vulnerabilities, such as increased expression of certain proteins and pathways, to selectively induce their death. Examples of senolytic drugs that have been studied in preclinical and clinical studies include dasatinib and quercetin, which act on the apoptotic resistance of SCs, as well as navitoclax and fisetin [65].

In animal studies, senolytic drugs have demonstrated various health benefits, including improvements in physical function, reduction of inflammation, and extension of both lifespan and healthspan. Human clinical trials are currently underway to investigate the efficacy and safety of senolytic drugs in treating ARDs. However, much research is still needed to fully comprehend the long-term effects of this drug class. The most well-known senolytics are pharmacological compounds that target proteins involved in apoptosis and the cell cycle. ABT263 (navitoclax) is a senolytic agent that acts on Bcl-2 and Bcl-xL and has been studied in mouse models of ARDs, such as atherosclerosis and neurodegenerative diseases [66]. Treatment with ABT263 decreases the senescence rate in tissues by inducing the apoptosis of SCs and alleviating the symptoms of diseases. On the other hand, UBX0101 acts by disrupting the interaction between MDM2 and p53, similarly inducing apoptosis in SCs [67]. MDM2 is involved in the ubiquitination of p53 and its proteasome degradation. Blocking their interaction prevents the elimination of p53, leading to the onset of a senescent status [67]. UBX0101, a drug targeting this interaction, is currently under clinical phase 2 studies in patients with osteoarthritis [39]. HSP90 is identified as a novel senolytic drug, functioning in the apoptosis process similarly to ABT263 [60]. 

Senescence has been regarded as an evolutionary adaptation, allowing the reduction of the risk of cancer development, and spreading by halting the proliferation of damaged cells. Therefore, when discussing senolytic agents and their potential application in clinical practice, it is crucial to consider the nonspecific action of these agents on SCs, which may also play a positive role in limiting cancer and facilitating mechanisms associated with fibrosis and wound healing [31]. A potential application of senolytic drugs in clinical practice requires the establishment of a standardized process, specifically targeting SCs to also avoid side effects associated with the action of senolytic agents on non-SCs. Therefore, the development of study models is crucial to understand the behaviour of SCs in physiological conditions, throughout the ageing process, and to gain a better understanding of how to effectively utilize senotherapeutic agents [22,61,65,68,69]. A senolytic can still be effective even if it eliminates only a significant fraction of SCs. It employs a “hit-and-run approach” with low doses, in contrast to anticancer therapies that use high doses to destroy malignant cells in a more hormetic process [23].

Using a priori knowledge of their molecular targets and mechanisms of action, compounds like dasatinib and quercetin were identified as potentially senolytics due to their predicted ability to transiently disable SCAP networks, allowing SCs to start the apoptosis process. Specifically, dasatinib, selected based on specific tyrosine kinase targeting, has been employed as a treatment against leukaemia and other crucial SCAP elements. Quercetin, found in strawberries, grapes, red wine, and tomatoes, was chosen for its targeting of members of the Bcl-2 family, hypoxia-inducible factor-1α, and specific nodes in the antiapoptotic PI3-kinase and p21 pathways.

Given their demonstrated restricted action on adipose and endothelial cells, the combination of dasatinib and quercetin (D + Q) was utilized in subsequent in vivo studies in mice and clinical trials. In a recent study by Hickson et al. [70], D + Q (1 µM + 20 µM) or a vehicle for 48 h was used to assess their effectiveness in a human omentum adipose tissue specimen, considering the relationship between obesity and the accumulation of SCs. This treatment did not affect the SCAPs but reduced the expression of senescent markers, including p16 and SA–β–Gal. Conversely, D + Q caused a reduction in SASPs in the conditioned medium after 24 h from the 48 h treatment. D + Q also impacted the proliferation and function of adipose tissue, regulating crucial proteins involved in tissue homeostasis [70].

Moreover, treating young mice with D + Q at the time of SC transplantation for 3 days mitigated the deteriorations in walking speed, hanging endurance, and grip strength that occurred one month later in vehicle-treated SC-transplanted mice, suggesting that D + Q treatment was sufficient to prevent the physical dysfunction caused by SCs. Similar results were observed with prolonged treatment. A clinical trial [69] was conducted on patients with diabetic kidney diseases, administering 3 days of D + Q (100 and 1000 mg). Eleven days after the treatment, the SC burden was reduced, with decreasing levels of p21 and p16 expression cells, SA–β–Gal, and progenitors with limited proliferative capacity. Additionally, macrophages of adipose tissue decreased, as did SASPs in adipose tissue and skin specimens. The results confirm the hit-and-run theory, emphasizing the positive outcomes achievable with senolytic applications.

This theory is grounded in the idea that a localized action of senolytic agents can be advantageous for addressing alterations associated with cellular senescence, preserving the beneficial properties of SCs. Prolonged treatment, on the other hand, might disrupt the positive impact of senolytic agents, leading to potential side effects. Additionally, periodic or cyclic treatment with senolytics may promote positive effects to a lesser extent. This concept aligns with the notion of the hormetic action of certain substances, wherein positive effects are observed at lower doses [58].

Furthermore, D + Q alleviated physical dysfunction in patients with idiopathic pulmonary fibrosis, a progressive and fatal disease associated with cellular senescence. The accumulation of SCs has also been observed in aorta hyporeactivity and atherosclerosis, suggesting a potential application of D + Q in these pathologies as well [70]. In another clinical trial, extended administration of D to patients with systemic sclerosis appeared to reduce the SASP and other senescence markers in skin biopsies [70].

As mentioned earlier, there is a limitation in the application of senolytic drugs to target SCAPs. Different SC types utilize different SCAPs or even redundant combinations of SCAPs to evade apoptosis. This implies that agents targeting a single SCAP may only eliminate a subset of SCs. One drug capable of acting on SCAPs is AP20187. In INK-ATTAC mice, it activates the suicide protein ATTAC. In these mice, the expression of p16 is related to that of activated caspase 8 (FKBP-Casp8) and a reporter gene (green fluorescent) protein [33]. The administration of senolytic drugs, such as AP20187, has been shown to alleviate a variety of conditions caused by SCs implanted in the animal knee, ranging from arthritis to vasculopathy and the risk of atherosclerotic plaque formation. These drugs inhibit the expression of p16, leading to the activation of RB and inducing the expression of caspase 8 dimers, thereby promoting the apoptosis of SCs [33].

In recent years, two well-known antibiotics, azithromycin and roxithromycin, have been discovered to possess senolytic activity targeting SCs. Additionally, curcumin has been identified as having the ability to act on sirtuins and the AMPK pathway [60]. This underscores the importance of broadening the search for senolytic agents to include drugs commonly used for senescence-related or non-senescence-related diseases, such as antibiotics, or natural products like curcumin (see below, Nutritional Senotherapeutics).

Experiments with mice showed that targeting p16^Ink4a^ with a senolytic agent increased the lifespan of the mice by 30% [71]. The expression of p16^ink4a^ seems to be related also to the incapacity of wound healing and the alteration of organs with ageing, such as liver fibrosis, lung injuries, and bone frailty [72]. Two studies on mice, one utilizing a p16-3MR mouse, a model characterized by a p16 promoter associated with reporter proteins and with a sensitive region to ganciclovir, which permits the killing of SCs [73], and the other a p16^Ink4a^ reporter mice, in which a histone H2B-green fluorescent protein is expressed with the *p16INK4a* gene product in the murine Cdkn2a locus of a bacterial artificial chromosome, to provide multiple copies of a stable fluorescent protein, showed that the treatment with senolytics delayed the wound healing [28,74]. Furthermore, the use of senolytics that target p16, like dasatinib and quercetin, could affect also the non-SCs, delaying tissue reparation. In contrast with these findings, a study about the reparation of bone fracture in mice determined that the use of senolytics agents, which act on p21, caused an acceleration in the capacity of bone healing [75]. The same results were obtained on models of lung injuries in mice. The conditioning of the cell medium with SASPs has been demonstrated to induce the expression of stem cell markers and genes that induce the regeneration capacity of mice keratinocytes [76]. Therefore, one of the considerations about the efficiency of the use of senolytics on the healing capacity is that the presence of few SCs induced by the continuous administration of senolytics impaired the capacity of healing of injuries in mice, while higher levels, due to intermitting administration of senolytics, drive the expression of SASPs, and thus the increased capacity to fit the damages [68]. Another type of variability is evident when using senolytics like dasatinib, quercetin, and fisetin, because the efficacy differs when applied to different cell types, like preadipocytes, mesenchymal stromal cells, or senescent human umbilical vein endothelial cells. Another consideration in using senolytic agents that target SCAPs is the potential to affect nodes within these pathways, such as the one formed by Bcl-2. In such cases, there is an increased likelihood of undesired effects related to dosage and unintended action against non-SCs, potentially exacerbating side effects like neutropenia and thrombocytopenia. It has been observed that using low doses may heighten side effects, while high doses impact a greater number of SCs, influencing the involved nodes [22]. 

In the same way, the use of SRC/tyrosine kinase inhibitors or flavonoids has been shown to take action on the proapoptotic capacity [22].

### 3.2. Senomorphics

Senomorphics act on SASPs and contribute to the clearance of SCs. They encompass inhibitors of IkB kinase (IKK) and NF-κB, scavengers of free radicals, and the JAK pathway [77]. Rapamycin is one of the agents identified as senomorphics. It targets mTOR and has been observed to extend lifespan in animal models while reducing the occurrence of cancer, heart attacks, dementia, and immune dysfunction. In humans, rapamycin enhances the response to influenza vaccination [12]. Ruxolitinib, a JAK1/2 inhibitor, diminishes age-related alterations in adipose tissue, insulin resistance, and stem cell dysfunction in ageing animal models [12]. Other potential senomorphic agents include metformin, the primary drug for diabetes, which also acts at the metabolic level and has shown positive effects on longevity in animal models, with ongoing human studies [12].

Similar to other senolytic agents, the treatment with SASP inhibitors needs to be intermittent to prevent off-target effects associated with the action of these drugs in other pathways. For instance, rapamycin may induce insulin resistance as an undesirable side effect [12]. 

In the study of senomorphic agents, the type of SASPs produced by cells plays a role, and this depends on the stimulation of SCs, the hormonal milieu, as well as the concurrent administration of drugs such as glucocorticoids, metformin, or JAK1 and 2 inhibitors [12]. The primary targets of senomorphic agents include the NF-κB pathway and mTOR, which is involved in the regulation of NF-κB by IL-1α. IL-1α, in turn, regulates the production of SASPs, such as IL-6 and IL-8, and modulates the DNA-binding activity of C/EBP β. Other targets include MAPKs and MAPK-activated protein kinase 2, activated by p38-MAPK, thereby promoting NF-κB activation. TNF-α, through its action on AKT and STAT3, contributes to the activation of NF-κB, leading to the amplification of SASP production [61].

Using a senomorphic agent that acts nonspecifically on SASPs may result in incomplete action, failing to neutralize all produced SASPs. Limitations in studies on this type of senotherapeutics pertain to the lack of specificity of action. SCs are not the exclusive producers of inflammation mediators or other molecules, defined as SASPs, so the administration of these agents could also affect non-SCs, including innate and adaptive immune cells. Another issue involves the necessity for prolonged administration of these agents. While senolytics can be administered in a hit-and-run approach, senomorphic agents require continuous administration to prevent the spread of SCs. This could lead to an increased risk of side effects, related to the suppression of positive properties linked to cellular senescence and effects on non-SCs. A potential solution might come from using engineered antibodies targeting only the SASPs of SCs.

### 3.3. Immune Therapies

#### 3.3.1. Immune Cell Response

Normally, SCs, through their secretory activity represented by SASPs, release chemokines and factors that facilitate the recruitment of immune system cells, such as macrophages, neutrophils, NK cells, and T cells. For instance, SCs expressing p53 can recruit NK cells by releasing the chemokine CCL2. However, some SCs, especially with ageing, can evade immune system activity, promoting inhibition. This occurs, for example, with the upregulation of CD47, which acts as an inhibitor against macrophages, or in the balance between the overexpression of HLA-E and the shedding of the NKG2D ligands, MHC class I chain-related protein (MIC)-A and MIC-B [25]. Furthermore, the ability of the immune system to eliminate SCs decreases with age due to immune ageing [27,78].

In the context of the immune response to SCs, a potential role in senolytic therapy is played by T cells with cytotoxic activity. Among these, cytotoxic CD4+ cells that increase in the peripheral blood of the oldest centenarians seem of particular interest, as it has been demonstrated that the number of these cells correlates positively with the number of senescent fibroblasts in the skin of older women. This appears to be due to the action of CXCL9, expressed by senescent fibroblasts, which attracts cytotoxic CD4^+^ T cells that recognize the gB antigen of cytomegalovirus (HCMV) presented by HLA-II. Also, CD8+ cells, whose effector memory subset recognizing HCMV antigens significantly increases in the oldest centenarians, appear to play a role in the clearance of SCs, although they can be blocked by the PD-1/PDL-1 checkpoint. In models, monoclonal antibodies against PD-1 would optimize their senolytic activity. However, it is logical to hypothesize that the anti-HCMV and anti-SC properties of these subsets contribute to achieving longevity [79,80,81].

Recently, a significant age-related increase in CD56^+^CD16^+^ NK cells has been demonstrated, with the highest values observed in the oldest centenarians [82]. It is intriguing to note that NK cells have been suggested to play a crucial role in the immune surveillance of SCs, as their activating receptors can promptly recognize stressed cells, making NK cells unique in their role as sentinels of SCs (refer to the paragraph below on NK and CAR NK cells). Moreover, it has been shown that the percentage of circulating CD56^+^CD16^+^ NK cells is also negatively correlated with the onset and staging of colorectal cancer [83]. Again, it is logical to infer that both these properties contribute to achieving longevity.

#### 3.3.2. CAR-T Cells

One approach to target SCs involves the use of T cells engineered to specifically recognize them. This has given rise to the concept of employing chimeric antigen receptor (CAR) T cells as senolytic agents. CAR-T cells are T cells that have been genetically engineered to express artificial receptors with specificity for a particular antigen. Originally developed for the treatment of B-cell neoplasia, this therapy has been extended to clinical practice, including applications for solid cancer [84]. 

The initial step in developing CAR-T cells for SCs is to identify markers that are specifically expressed on the cell membrane of SCs. One potential target for this purpose is represented by NKG2D, a molecule involved in the senescent phenotype [85].

By analyzing RNA sequencing datasets from three senescence models (therapy-induced senescence in mouse lung adenocarcinoma cells, oncogene-induced senescence in mouse hepatocytes, and culture-induced senescence in mouse hepatic stellate cells), Amor et al. [62] identified plasminogen activator, urokinase receptor transcripts, which encode the urokinase-type plasminogen activator receptor (uPAR) protein. uPAR was identified as a senescence marker, and its cleaved form, suPAR, was recognized as a component of SASPs, expressed concurrently with other senescence markers such as IL-6 and p16. uPAR plays a role in promoting extracellular matrix degradation during fibrinolysis, wound healing, or tumorigenesis, and it serves as a signalling receptor that enhances the motility, invasion, and survival of cancer cells.

The development of T cells engineered to recognize murine uPAR in human lymphoma cells and in vivo in hepatocarcinoma models demonstrated that CAR-T therapy can reduce the number of SCs and related SASPs, thereby improving the clinical situation of the studied animal models. uPAR expression and serum suPAR levels have been associated with various human diseases, including diabetes, atherosclerotic plaque formation, and liver and lung fibrosis, as well as intraepithelial lesions in pancreatic tumours [62].

The establishment of a mouse model of liver fibrosis treated with CAR-T cells engineered to recognize uPAR revealed the effectiveness of this treatment in diseases related to the accumulation of SCs in tissues, such as liver fibrosis. Moreover, it was observed that prolonged treatment was well-tolerated, while the use of excessive doses resulted in a phenomenon similar to the cytokine release syndrome observed in treatments for human malignancies, which could be resolved with cytokine inhibitors. This opens up new possibilities for the treatment of ARDs, and most importantly, raises questions about the need to identify increasingly specific markers for SCs to avoid the adverse effects associated with the administration of senolytic agents and to determine the optimal timing for treatment [62]. 

The potential use of engineered T cells raises the question of manipulating the immune system to selectively eliminate SCs, akin to the application of monoclonal antibodies or CAR-T cells in cancer treatment. However, manipulating the immune system encounters a challenge in ARDs where the accumulation of SCs is linked to or results from the immunosenescence process. Therefore, in a cascading effect, it is crucial to identify the key factor that can intervene to halt the sequence of interconnected and often independent events leading to the health deterioration associated with ageing.

#### 3.3.3. NK and CAR NK Cells

Together with CAR-T cells, another therapy developed for leukaemia and solid cancers, but applicable to senescence, is represented by CAR NK cells [63]. This therapy offers advantages compared to CAR-T, owing to the innate characteristics of the immune response mediated by NK cells towards T cells and the lower incidence of rejection episodes. CAR NK cells, unlike CAR-T cells, possess a nonspecific receptor, mainly NKG2C, coupled with DAP10/12 and a co-stimulatory domain, acting directly on cells through antibody-dependent cell-mediated cytotoxicity (ADCC) or cross-reacting with other immune cells [63].

Among the markers specifically expressed by SCs is CD26, also known as dipeptidyl peptidase 4 (DPP4). Its expression has suggested the possibility of selectively eliminating SCs using immune cells directed against it. The main mechanism by which SCs are eliminated by the immune system involves ADCC, raising the possibility that these cells can be targeted by NK cells or CAR NK cells. In a study using WI-38 human diploid fibroblasts, the potential to selectively target CD26 for eliminating SCs in a model of liver fibrosis was investigated. Silencing the gene encoding DPP4 in these cells resulted in a reduction in the senescent phenotype, with decreased expressions of p21, p53, and p16, along with increased expression of sirtuin1. Notably, DPP4 expression was found to be higher in peripheral blood samples from older subjects (78–88 years of age) compared to younger subjects (27–36 years of age), and this expression was more associated with monocytes and lymphocytes. Using antibodies directed against DPP4 and coculturing with human NK cells, the viability of SCs was reduced by 40% compared to proliferating cells. DPP4 is related to the cell surface expression of caveolin 1, which is involved in the activation of NF-κB and the production of SASPs. Blocking DPP4 could potentially affect SASP production. DPP4 is best known as a protease that inactivates two hormones named incretins (glucose-dependent insulinotropic peptide and glucagon-like peptide-1), which are involved in the rapid release of insulin from pancreatic β cells after a meal. Therefore, understanding the contribution of DPP4 to senescence onset and how anti-incretin treatment acts on SCs would be interesting [86].

The mechanism of immunosurveillance involving NK cells also involves the expression of another marker on SCs, NKG2D. It was observed that the knockdown in mice liver fibroblasts leads to increased liver fibrosis. ULBP1-6, MICA, and MICB are the ligands of NKG2D, playing a role in the elimination of damaged cells by NK cells when cells undergo senescence induction. IMR-90, WI38, and BJ normal human fibroblasts induced to senescence developed increased levels of NKG2D ligands in vitro. Blocking the binding between NKG2D and their ligand, expressed on the surface of SCs, impaired the elimination of those cells. These findings were also demonstrated in vivo on mouse models of liver fibrosis by inducing the silencing of the expression of the *NKG2D* gene and evaluating the immune clearance of SCs, resulting in increased liver fibrosis [87]. In other models, the deletion of NKG2D enhanced the rate of stellate hepatic cells, and thus, fibrosis. The role of NK cells in clearing tumour SCs was also evaluated as an indirect effect of cytokines produced by activated NK cells on macrophages, leading to the elimination of SCs [88].

#### 3.3.4. Monoclonal Antibodies

Another approach involves the development of monoclonal antibodies targeting SC markers. In a mouse model of age-related bone loss, grancalcin, a calcium-binding protein, was associated with macrophages and neutrophils, particularly inhibiting osteogenesis and inducing adipogenesis, both considered senescence-linked phenomena. The mechanism of action of grancalcin involves a molecular pathway, with the Plexin B2 protein playing a role. Administering monoclonal antibodies against grancalcin demonstrated an improvement in the animal bone condition without affecting the production of inflammatory cytokines, achieved by blocking the binding of grancalcin to Plexin B2 [89].

**Figure 1:**
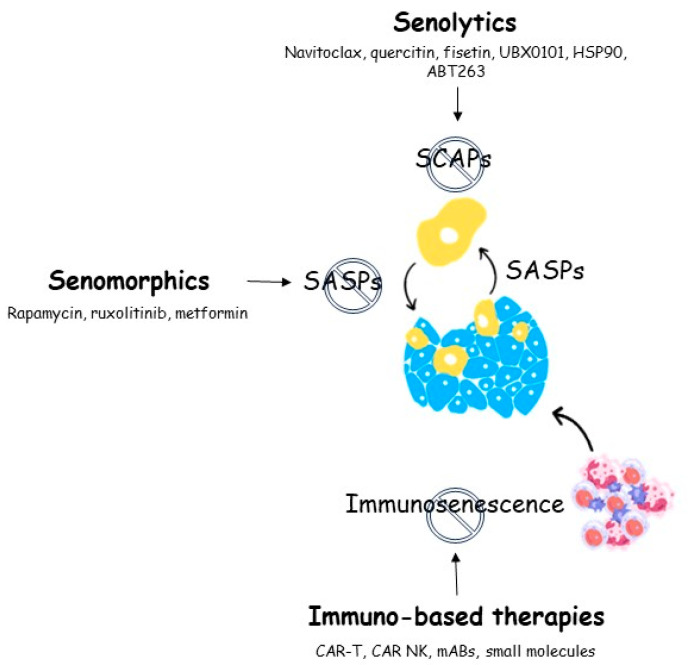
Senotherapeutic agents and their targets.

Figure 1 summarizes the mechanisms of senotherapeutics.

## 4. Nutritional Senotherapeutics

Nutrition also plays a crucial role in mitigating unsuccessful ageing. There are models of successful ageing represented by two populations, one of which is the Okinawan population. The percentage of individuals who have reached or are nearing 100 years old is remarkably high. The combination of lifestyle, characterized by a low-carbohydrate and protein-rich diet, especially from fish, along with environmental factors, has designated it as a Blue Zone and a global model. The Okinawan diet emphasizes calorie restriction (CR), which appears to be the foundation of longevity. This dietary approach has been tested on animal models, yielding mixed results. It was observed that there are not only beneficial effects, as prolonged treatment leads to the loss of bone and muscle mass, along with asthenia. Hence, the Okinawan pattern is certainly not solely attributed to the diet but is a combination of various factors, both genetic and environmental, contributing to the population’s exceptional longevity [90]. The role of CR in counteracting cellular senescence depends heavily on the high phytochemical content of this type of diet. In fact, the Okinawan population’s high consumption of plant foods, rich in polyphenols, thanks to the climate that favours their richness, results in a powerful anti-inflammatory and antioxidant effect in this population, which is echoed in the CR model. Polyphenols act on several key pathways, such as nuclear factor erythroid 2-related factor (Nrf)-2, FOXO, and IGF-1 [91]. Okinawan traditional foods, such as bitter melon (low in caloric density, high in fibre and vitamin C), contribute to the regulation of glucose levels. Tofu, the main protein source, is involved in the regulation of the sirtuins-FOXO pathway and anti-inflammatory regulation. Studies on CR and the regulation of cellular senescence indicate that it acts by limiting the source of damage that induces senescence, thus reducing oxidative stress and cellular inflammation, or by inducing the elimination of damaged cells through autophagy. The first mechanism occurs through action on Nrf-2 pathways, FOXO, and by regulating the expression of sirtuins. The second mechanism involves the modulation of mTOR. Similar to the Okinawan diet, there is modulation of IGF-1 expression and regulation of the pathway in which it is involved [92]. Some of the phytoconstituents found in the Okinawan diet, such as resveratrol (which activates sirtuins and FOXO-3, linked to the IGF-1 pathway), genistein (contained in tofu, acting on FOXO-3), isoflavone (acting on pathways involved in apoptosis in human tumour models), and curcumin (acting on the NF-κB pathway), are associated with the concept of longevity. Consequently, one could describe a senomorphics-oriented action of some phytoconstituents most represented in the Okinawan diet and CR, while others, such as flavonoids and isoflavones, appear to act in a senolytic sense.

The same holds true for the Mediterranean diet, which proves effective at reducing the occurrence of ARDs. The success of this diet is mainly attributed to the inclusion of foods rich in polyphenols, similar to the Okinawan diet, although the micronutrient intake is higher for the Okinawan diet compared to the Mediterranean one (58% versus 42%) [91,93]. On the nutritional front, major senomorphic agents identified include resveratrol, kaempferol, apigenin, and epigallocatechin gallate (EGCG). The first three, excluding EGCG, act on NF-κB, regulating inflammation and oxidative stress, and impact the Nrf-2 transcription factor, which is activated upon NF-κB inhibition. On the other hand, EGCG suppresses senescence by influencing PI3k/AKT/mTOR and inhibiting AMPK activation (Table 2). Senolytics studied include quercetin, fisetin, piperlongumine, and curcumin. In vivo studies and several clinical trials involving these compounds have been initiated. For example, many clinical trials have been set up to study the combination of the compounds with senotherapeutic activity just mentioned. For instance, the trial NCT04994561 is studying the combination of resveratrol, quercetin, and fisetin, and is still in phase I. The AFFIRM-LITE trial, on the other hand, is studying the effects of quercetin and is ongoing [61]. Moreover, some of these compounds, such as resveratrol, also affect the metabolism of SCs by enhancing mitochondrial activity and reducing glycolytic metabolism [61].

## 5. Nanoparticles

Nanoparticles (NPs) activated by β–Gal have been shown to eliminate SCs both in vitro and in vivo [64]. By studying the surfaceome (all plasma membrane proteins that have at least one amino acid residue exposed to the extracellular space) of SCs, potential epitopes have been identified using mass spectrometry. These epitopes are then utilized to design monoclonal antibodies, which are linked to drugs specifically cytotoxic to SCs through special linkers. This represents the development of NPs designed at the molecular level to target SCs. This approach was demonstrated in kidney carcinoma cell lines (Ejs) induced to senescence after gene silencing. The expression of the *β-2-macroglobulin* gene (B2M) was found to be strongly associated with the upregulation of p53 and, under certain stress conditions, also with p16. An antibody-drug conjugate was constructed to target B2M in SCs. Bound to duocarmycin, a small molecule involved in cancer therapy, it selectively induces SC death by releasing duocarmycin into them, without significantly affecting the survival of proliferating control cells. This is supported by data showing cell death in the HCT116 model of chemotherapy-induced senescence, a well-studied example of stress-induced senescence [94].

## 6. Conclusions

The pursuit of drugs that combat ageing at the systemic level and address molecular ageing has always captivated both the scientific community and society. Anti-ageing therapies are not aimed at halting the inevitable and essential ageing process but rather at ensuring that individuals reach older ages in the best possible state of health. The development of pharmacological strategies relies on identifying markers of cellular and molecular ageing—proteins expressed to a greater extent by cells undergoing the ageing process.

The presence of SCs in tissue contexts contributes to the typical dysfunctions of ARDs. Eliminating these cells in animal models and certain clinical trials has underscored the potential for systemic improvement in the health conditions of individuals affected by senescence-related diseases. Like many therapies in development, the key lies in identifying specific and safe targets and establishing standardized guidelines for treating cellular senescence. This approach ensures the initiation of multiple clinical trials based on well-established markers. 

It is intriguing to note that data on the immune response to senescent cells (SCs) suggest that control by lymphocyte subsets increased in oldest centenarians contributes to achieving extreme longevity. In any case, the use of techniques already employed in cancer therapy, such as CAR cells and monoclonal antibodies, expands the range of potential methods to be employed.

## Figures and Tables

**Table 1:** Senotherapeutic agents, their targets, and effects on SCs.

Senolytic Compounds		
Actions: induce apoptosis of SCs disabling SCAPs or other molecular signals involved in SCs rising	Examples: HSP90, dasatinib, quercetin, fisetin, curcumin	[60,61]
Positive effects	Reduce the amount of SCs and tissue-related dysfunction	
Negative effects	Reduction in positive effects of SCs (anticancer, antifibrotic, embryogenetic)	
**Senomorphic Compounds**		
Actions: inhibit senescence markers (i.e., NF-κB, IKK, JAK) and SASPs	Examples: rapamycin	[60]
Positive effects	Low impact on positive aspects of SCs; reduction in the paracrine role of SASPs on neighboured cells	
Negative effects	The necessity of continuous administration; the possibility of side effects	
**Immuno-Based Approaches**		
Actions: improve immune response toward SCs	Examples: CAR-T cells, NK cells, monoclonal antibodies	[25,62,63,64]
Positive effects	Targeting specifically SCs; impact directly or indirectly on SCs, without affecting their positive actions	
Negative effects	It is necessary to know specific markers and act in specific sites to avoid side effects	

**Table 2:** Nutritional senotherapeutics.

Nutritional Senotherapeutic	Effects	References
Senomorphics		
Resveratrol	Inhibits NF-κB and activates Nrf-2; increases OXPHOS and SIRT1 activation; acts on FOXO and IGF pathway	[61,91]
Genistein	Regulation of FOXO3	
Isoflavone	Modulation of apoptosis pathways	
Kaempferol	Acts on inhibition of NF-κB through IRAK1/IKB-α	
Apigenin	Acts on inhibition of NF-κB through IL-1rα modulation acting on IRAK1/p38MAPK	
EGCG	Inhibition of AMPK activation through the modulation of AKT/PI3k/mTOR signalling pathway. Inhibition of ROS, SASPs, NF-κB, and COX	
Fisetin	Antioxidant and anti-inflammatory action through modulation of NF-κB and Nrf-2	
Curcumin	Anti-inflammatory activity on NF-κB and antioxidative effects on Nrf-2.	
Piperlongumine	Antioxidant activity inhibiting ROS production	

## Data Availability

No new data were created or analyzed in this study. Data sharing is not applicable to this article.
